# Mayaro Virus Induction of Oxidative Stress is Associated With Liver Pathology in a Non-Lethal Mouse Model

**DOI:** 10.1038/s41598-019-51713-9

**Published:** 2019-10-25

**Authors:** Camila Carla da Silva Caetano, Fernanda Caetano Camini, Letícia Trindade Almeida, Ariane Coelho Ferraz, Tales Fernando da Silva, Rafaela Lameira Souza Lima, Mayara Medeiros de Freitas Carvalho, Thalles de Freitas Castro, Cláudia Martins Carneiro, Breno de Mello Silva, Silvana de Queiroz Silva, José Carlos de Magalhães, Cintia Lopes de Brito Magalhães

**Affiliations:** 1 Postgraduate Program of Biological Science, Biological Sciences Research Center, Universidade Federal de Ouro Preto, Ouro Preto, Minas Gerais Brazil; 2 Biological Science Departament, Universidade Federal de Ouro Preto, Ouro Preto, Minas Gerais Brazil; 3 Clinical Analysis Departament, Universidade Federal de Ouro Preto, Ouro Preto, Minas Gerais Brazil; 4 Postgraduate Program of Biotechnology, Biological Sciences Research Center, Universidade Federal de Ouro Preto, Ouro Preto, Minas Gerais Brazil; 5 Department of Chemistry, Biotechnology and Bioprocess Engineering, Universidade Federal de São João del-Rei, Ouro Branco, Minas Gerais Brazil

**Keywords:** Structural biology, Virology

## Abstract

*Mayaro virus* (MAYV) causes Mayaro fever in humans, a self-limiting acute disease, with persistent arthralgia and arthritis. Although MAYV has a remerging potential, its pathogenic mechanisms remain unclear. Here, we characterized a model of MAYV infection in 3–4-week BALB/c mice. We investigated whether the liver acts as a site of viral replication and if the infection could cause histopathological alterations and an imbalance in redox homeostasis, culminating with oxidative stress. MAYV-infected mice revealed lower weight gain; however, the disease was self-resolving. High virus titre, neutralizing antibodies, and increased levels of aspartate and alanine aminotransferases were detected in the serum. Infectious viral particles were recovered in the liver of infected animals and the histological examination of liver tissues revealed significant increase in the inflammatory infiltrate. MAYV induced significant oxidative stress in the liver of infected animals, as well as a deregulation of enzymatic antioxidant components. Collectively, this is the first study to report that oxidative stress occurs in MAYV infection *in vivo*, and that it may be crucial in virus pathogenesis. Future studies are warranted to address the alternative therapeutic strategies for Mayaro fever, such as those based on antioxidant compounds.

## Introduction

*Mayaro virus* (MAYV) is an arbovirus member of the *Alphavirus* genus and *Togaviridae* family. Despite little known by the population, Mayaro fever is an ancient disease. The first incidence of this virus was reported near Mayaro town in Trinidad and Tobago in 1954, where it was originally isolated from the patient’s blood, representing febrile illness of short duration^1^. Since then, Mayaro fever has been reported in several countries: Brazil, Peru, Suriname, French Guiana, Guyana, Venezuela, Colombia, Ecuador, Panama, Bolivia, Costa Rica, Guatemala and Mexico^2–9^. In Brazil, since its first isolation in 1955, the MAYV has been found mainly in the northern region. The virus is endemic in the Amazon region, where outbreaks were reported^10–13^. A majority of MAYV infections in humans occur in people who visit the forests frequently; however, in the past few years, these infections have been reported in urban/periurban areas, indicating the potential urbanization of Mayaro fever in Brazil^13–16^.

The Mayaro fever symptoms are similar to other arboviruses such as Dengue (DENV), Chikungunya (CHIKV) and Zika (ZIKV), including rash, fever, headache, myalgia, retro-orbital pain, diarrhea and long-term chronic arthralgia (more associated with CHIKV), leading to a disabling morbidity^17,18^. Due to the general nature of the clinical manifestations, it has been difficult to diagnose these infections. Thus, Mayaro fever, often masked by symptoms similar to other diseases, can be misdiagnosed with other arboviruses, which are endemic in common areas^15,19^.

Despite the outbreaks and spread of Mayaro fever into new locations, few studies are available on the cellular and molecular mechanisms of the disease and its pathogenesis. Thus, it is essential to elucidate the mechanisms involved in the pathogenesis of MAYV disease, as it may lead to severe health issues in near future^20^. Oxidative stress plays a pivotal role in pathogenesis of viral diseases^21–26^. Therefore, the oxidative stress can be interpreted as a disruption/dysregulation of redox control caused due to increase in oxidants reactive species and/or a reduction in the antioxidant system^27^. Reactive oxygen species (ROS) are reactive atoms or molecules generated by physiological or pathological processes^28^. Their abundance can cause cellular damage leading to the loss of integrity and functionality^29^.

Acting concomitantly with ROS, the antioxidant defense mechanism comprises enzymatic systems including superoxide dismutase (SOD), catalase (CAT), glutathione peroxidase and non-enzymatic antioxidants including vitamin C, vitamin E, carotenoids, glutathione and flavonoids^30^. Since the details of MAYV pathogenesis remain unclear, and experimental models are essential in the research of alphaviruses^31^, the animal models have gained immense interest in studying MAYV infection. We have reported that MAYV infected HepG2 cells induces ROS production and significant oxidative stress. In addition, we observed an increase in the SOD and CAT activities and the total glutathione content, indicating an imbalance between ROS production and antioxidant cellular defenses^32^; however, the ability for MAYV-induced oxidative damage *in vivo* remains unclear. Thus, this study aimed to evaluate the involvement of oxidative stress on hepatic pathology in BALB/c mice infected with MAYV, thereby providing novel insights for understanding MAYV pathogenesis.

In our study, the infected mice developed weight loss, high levels of viremia, hepatic viral loads and a neutralizing antibody response. None of the infected mice died; however, the MAYV infection induced liver damage, as indicated by the increase in serum levels of aspartate and alanine aminotransferases (AST/ALT) and by significant increase of inflammatory cells in liver parenchyma. In addition, we determined several markers of oxidative injury (malondialdehyde, carbonyl protein, myeloperoxidase, and reduced versus oxidized glutathione ratio) and antioxidants (SOD and CAT) in liver. Our results revealed that MAYV induced significant oxidative stress in liver of infected animals, as indicated by an increase in MDA, carbonyl protein, myeloperoxidase (MPO) activity and decrease in reduced versus oxidized glutathione (GSH/GSSG) ratio. In relation to antioxidants, in general, a lower activity of SOD and CAT enzymes was observed in the liver of the infected animals soon after the infection. The findings of this study suggest that the redox unbalance may have been responsible for the oxidative damage in liver pathology of MAYV. Consequently, this event could essentially contribute to MAYV pathogenesis. These results, collectively, with our previous findings that antioxidant inhibits MAYV replication and attenuates MAYV-induce oxidative stress *in vitro*^33^, warrant future investigation on the use of antioxidants for preventing oxidative liver damage during Mayaro fever.

## Results

### MAYV is not lethal in BALB/c mice

**Figure 1:**
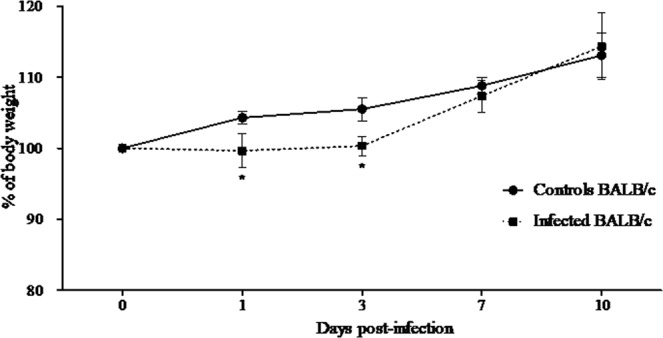
Percentage body weight in BALB/c mice infected with MAYV. The body weight of the control and MAYV-infected BALB/c mice were recorded. The percentages of mean weight relative to their initial weight were plotted from Day 0 to different days after inoculation (1, 3, 7 and 10). The data are expressed as percentage of weight gain and the results are expressed as the mean ± SEM (*n* = 9 per group).*p < 0.05 indicate significant differences compared with the control animals, Student’s t-test.

**Figure 2:**
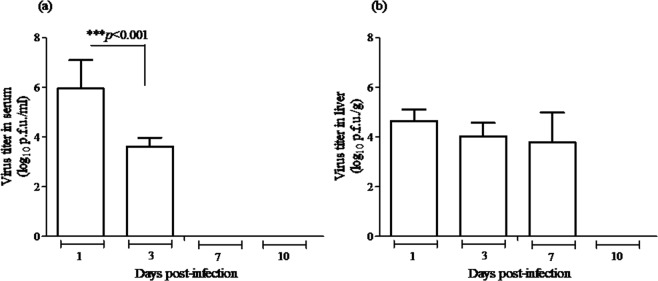
Virus titre in serum and liver of BALB/c mice infected with MAYV. (**a**) Mice inoculated with 10^5^ p.f.u. of MAYV were sacrificed, and then, on different days after infection, blood was collected, and the serum samples were used for viremia measurement. (**b**) Liver samples were homogenized, and virus was titrated. Serum and liver titres are expressed as log_10_ p.f.u./ml and log_10 _p.f.u./g of tissue, respectively. Absence of bars indicates that the virus was undetectable. Data are expressed as mean ± SEM (*n* = 9 per group) and results are from three independent experiments. Groups were compared using one-way ANOVA with Bonferroni’s post-test; significant *p*-values are depicted in the graphs.

About 3–4-week-old BALB/c mice were tested for susceptibility to subcutaneous infection with 10^5^ plaque-forming units (p.f.u.) of MAYV and all animals infected survived. To examine the clinical signs of infection, mice were monitored daily for 10 days and their weight was determined. MAYV-infected mice presented lower weight gain on Days 1 and 3 p.i. when compared to the control animals (Fig. 1). Importantly, no mice developed paralysis, rash or signs of swelling in the legs at any time during the observation period. Infected mice had detectable viremia on Days 1 and 3 p.i. and viral load in the liver on Days 1, 3 and 7 p.i. (Fig. 2a,b). To evaluate the humoral immune response developed against MAYV, infected animals of each day post-infection were tested for the induction of neutralizing antibodies. The sera were harvested and used for neutralization assays. On Days 7 and 10 p.i., the infected animals exhibited neutralizing antibody titre of 12,8000 and 25,6000 (NU/ml), respectively. Collectively, survival analysis, weight loss monitoring, and antibody neutralization titres provided evidence that BALB/c mice developed disease and survived infection by MAYV; however, the disease was self-resolving and the animals regained weight during the course of the infection. Furthermore, the viral infection was characterized in detail.

### MAYV infection results in liver pathology in BALB/c mice

**Figure 3:**
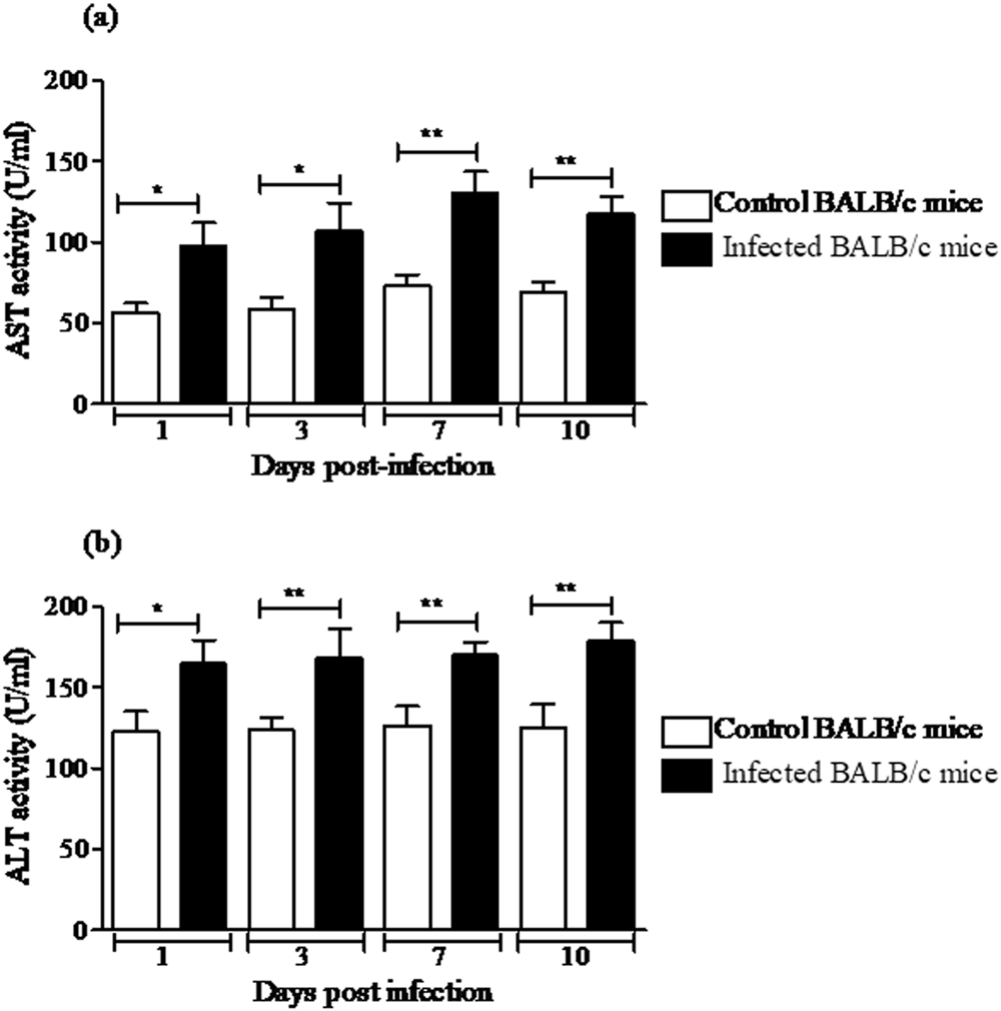
Serum levels of aspartate and alanine aminotransferases (AST/ALT). (**a**) AST activity. (**b**) ALT activity. Sera from control and MAYV-infected BALB/c mice were used to determine the AST and ALT activity levels on different days after inoculation. The data are expressed as the mean ± SEM (*n* = 9 per group). *p < 0.05 and **p < 0.01 indicate significant differences compared with the control animals, Student’s t-test.

**Figure 4:**
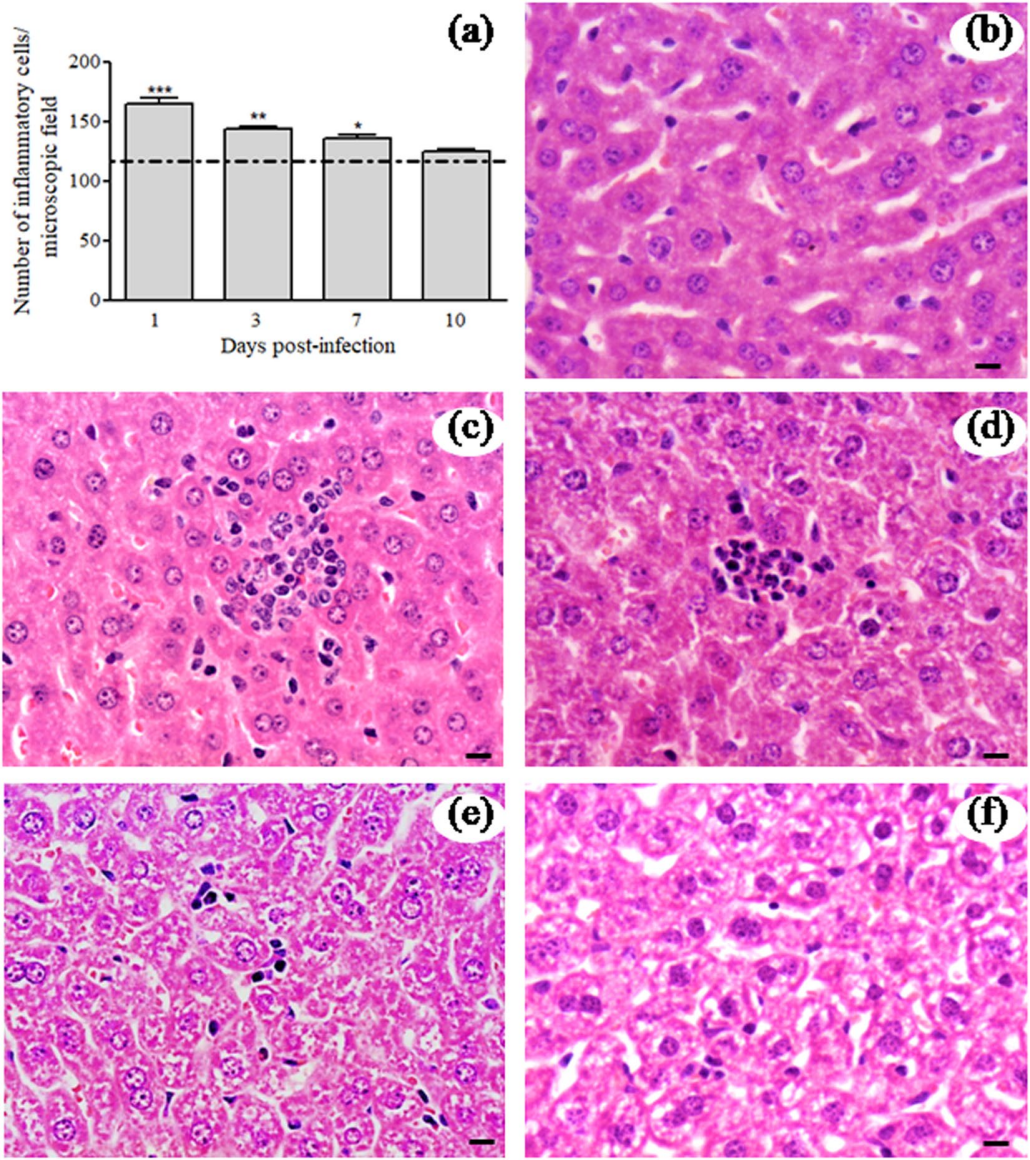
Histopathological analysis of liver tissues from control and MAYV-infected mice. (**a**) Morphometric measurements of inflammatory cells in the livers of BALB/c mice. The numbers of inflammatory cells in the liver sections of control and MAYV-infected BALB/c mice were enumerated on different days. The dashed line represents the mean number of cells quantified in hepatic histological sections of uninfected animals. (**b**) Controls for comparison are from liver sections mock-infected mice at 1 d.p.i. (**c–e**). Representative slides from MAYV-infected mice, in which the inflammatory cells were observed in infected animals on Days 1 (**c**), 3 (**d**) and 7 (**e**) p.i. Notably the absence of histological alterations in the control and infected animals on Day 10 p.i. (**f**). *, ** and *** indicate significant differences relative to the control group at *p* < 0.05, *p* < 0.01 and *p* < 0.001, respectively. To analyse the inflammatory cell count, two-way ANOVA analysis of variance was used followed by the Bonferroni’s post-test. Hematoxylin–eosin staining images. Black bars indicate 40 µm of magnification.

In order to efficiently characterize the hepatic effect of MAYV infection in BALB/c mice, levels of aspartate and alanine aminotransferases (AST/ALT) were determined in the serum. The mean AST and ALT levels were significantly increased in infected animals at all time-points tested (Fig. 3a,b). These results indicate that MAYV infection induced the disease with notable involvement of hepatic injury. Moreover, histopathological analysis of liver samples was conducted, and the results revealed alterations that were characteristic of the infection. Morphometric analysis revealed a significant increase in the number of inflammatory cells in the hepatic parenchyma of the infected animals on Days 1, 3 and 7 compared with that of the normal mice liver tissues (Fig. 4a). The histopathological alterations in the livers of infected mice were more evident on Day 1 p.i. (Fig. 4c), Day 3 p.i. (Fig. 4d) and on Day 7 p.i. (Fig. 4e) and were absent on Day 10 p.i. (Fig. 4f), when they were similar to the controls (Fig. 4b). The inflammatory infiltrate in the hepatic parenchyma of MAYV-infected mice showed a predominance of polymorphs cells, beyond lymphocytes and kupffer cells. Collectively, these results indicated that MAYV infection induced pathology and histopathological changes in liver of BALB/c mice.

### MAYV induces oxidative stress in liver of BALB/c mice

**Figure 5:**
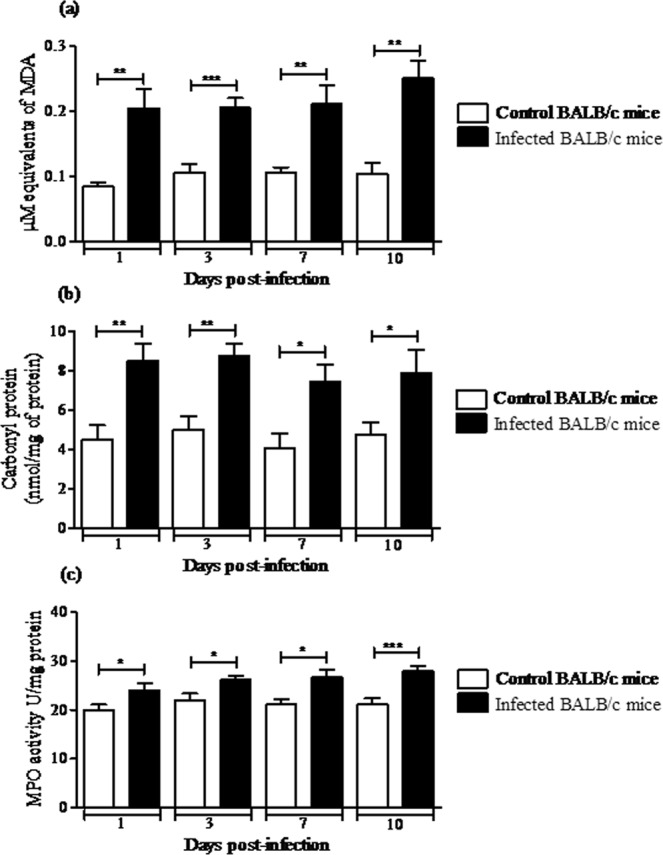
MAYV induces oxidative stress in liver of BALB/c mice. BALB/c mice were subcutaneously infected with 10^5^ p.f.u. of MAYV and 1, 3, 7 and 10 d.p.i. liver homogenate was used to measure the oxidative stress biomarkers MDA (**a**), carbonyl protein (**b**) and MPO activity (**c**). Mean ± SEM (*n* = 9 per group), and are representative of two independent experiments. *p < 0.05, **p < 0.01 e ***p < 0.001 indicate significant differences compared with the control animals, Student’s t-test.

To determine the intra-hepatic oxidative stress during MAYV infection, we measured the biomarkers of lipid peroxidation (MDA) and oxidative modification in proteins (carbonyl protein) in liver homogenate of control and MAYV-infected mice at 1, 3, 7 and 10 d.p.i. MAYV infection resulted in a significant increase of both biomarkers at all time-points tested compared with the control animals (Fig. 5a,b). Since MPO is found mainly in neutrophils and may contribute to disease pathogenesis via its enzymatic activity and production of oxidative radicals, we further evaluated its activity in the animal liver. As presented in the Fig. 5c, MPO activity was elevated in the liver of infected animals compared to the controls at all post-infection times. The GSH/GSSG ratio was also used as an indirect indicator of oxidative stress because its reduction indicates that more hydrogen peroxide (H_2_O_2_) is produced in the intra-cellular environment and that more GSH is being oxidized in GSSG in order to detoxify this oxidative species. Levels of total glutathione (T-GSH) and its reduced form (GSH) in liver of MAYV-infected mice on Days 1 and 3 p.i. were significantly lower when compared to the control group (Table 1). In contrast, on the same days, levels of the oxidized glutathione (GSSG) increased in the liver of infected animals (Table 1). The mean values GSH/GSSG were significantly lower in liver of MAYV-infected animals than the control group on Days 1 and 3 p.i. (Table 1). On Days 7 and 10 p.i., we did not observe any difference in T-GSH, GSH, GSSG and GSH/GSSG between the control and infected animals (Table 1).

**Table 1:** Glutathione values in liver of BALB/c mice infected with MAYV^a^.

	Day 1		Day 3		Day 7		Day 10	
	Control	MAYV	Control	MAYV	Control	MAYV	Control	MAYV
T-GSH	5.18 ± 0.14	3.84 ± 0.32**	5.38 ± 0.18	4.62 ± 0.28*	5.55 ± 0.31	6.27 ± 0.35	5.60 ± 0.20	6.10 ± 0.42
GSH	4.48 ± 0.04	3.04 ± 0.30**	4.73 ± 0.20	4.06 ± 0.28*	5.23 ± 0.22	5.78 ± 0.44	5.54 ± 0.40	5.00 ± 0.35
GSSH	0.60 ± 0.01	0.68 ± 0.02*	0.60 ± 0.03	0.71 ± 0.02*	0.54 ± 0.04	0.58 ± 0.04	0.62 ± 0.02	0.65 ± 0.06
GSH/GSSG	6.44 ± 0.38	4.57 ± 0.36**	7.86 ± 0.60	6.05 ± 0.50*	8.66 ± 0.65	9.80 ± 1.19	7.83 ± 0.49	7.68 ± 0.75

^a^Data are expressed with mean ± SEM (*n* = 9) and glutathione values are µmol/ml.

*p < 0.05 and **p < 0.0.

### MAYV infection alters the hepatic antioxidant in BALB/c mice

**Figure 6:**
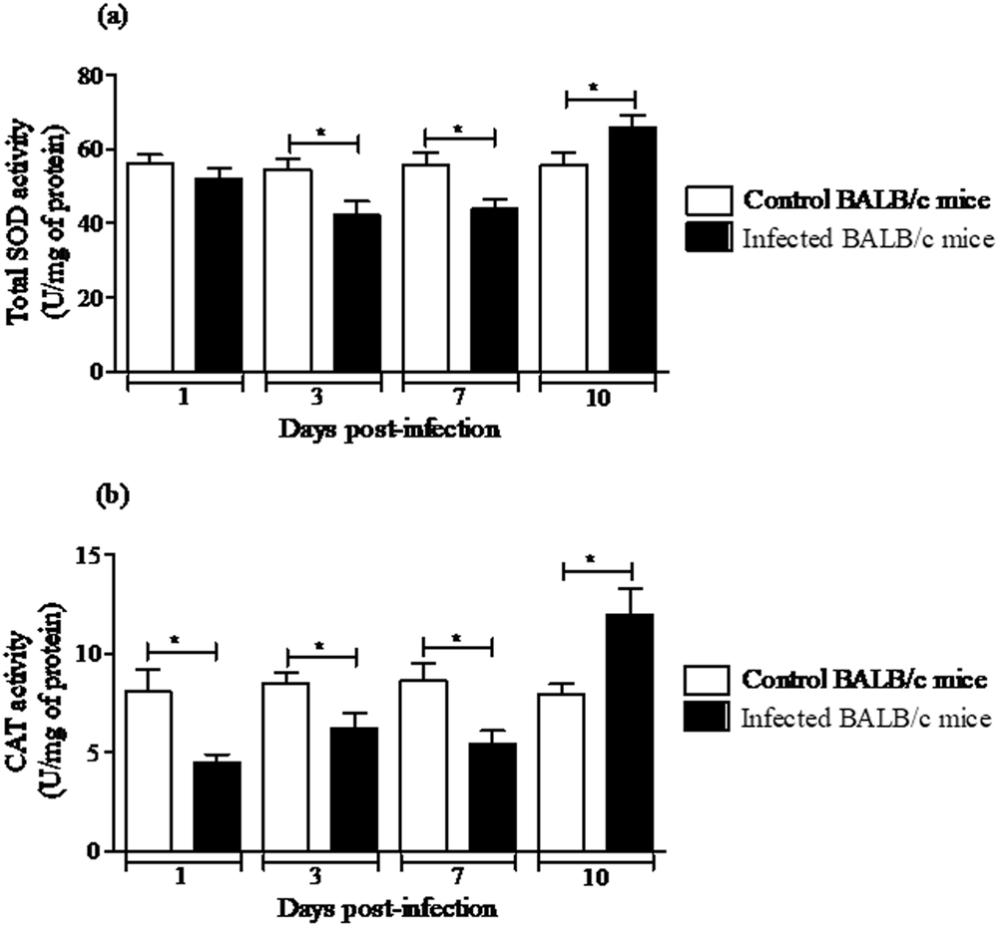
MAYV alters the enzymatic antioxidant defenses in liver of BALB/c mice. Liver homogenates were prepared from uninfected and MAYV infected mice for 1, 3, 7, and 10 d.p.i. to measure total SOD (**a**) and CAT activities (**b**). Results are expressed as the mean ± SEM (*n* = 9 per group) and are representative of two independent experiments.*p < 0.05 indicates significant differences compared with the control animals, Student’s t-test.

Since MAYV altered oxidative biomarkers and induced hepatic injury in liver of BALB/c mice, we investigated whether MAYV infection modifies the enzymatic antioxidant defences in the liver. The organ was homogenized and prepared from uninfected or infected BALB/c mice at 1, 3, 7 or 10 d.p.i. to measure the SOD and CAT enzymatic activities. In MAYV-infected mice, total SOD activity decreased significantly at Days 3 and 7 p.i. (22 and 21%, respectively), compared with the control mice. After 10 days, total SOD activity increased by 18% in the liver of infected animals (Fig. 6a). Compared with control mice, the activity of CAT decreased in the liver of infected animals on Days 1, 3 and 7 p.i. (50, 16 and 39%, respectively) and increased by Day 10 p.i. (68%; Fig. 6b).

## Discussion

Despite MAYV being an ancient virus and having a significant risk of emergency in the Americas, studies related to its pathogenesis and comprehension of its pathophysiology are limited. Therefore, the use of animal models to elucidate the mechanisms that contribute to this disease may provide a better understanding of the Mayaro fever^34^.

In terms of host mechanisms that address disease pathology, several studies have reported that the oxidative stress induced by virus can affect several aspects for the disease, demonstrating that inflammatory process and the stress response are important factors in the viral pathogenesis, thereby contributing to the severity of the disease^23,26,35–40^.

In the present study, to better characterize MAYV pathogenesis, we evaluated the markers of oxidative injury and antioxidants components in liver of BALB/c mice after MAYV infection. We chose the liver as the target organ because it is considered an important site of replication for *Alphavirus*, which facilitates their proliferation in the infected organism^41–43^. Furthermore, we have previously demonstrated that MAYV is able to replicate in human liver carcinoma cells (HepG2), thereby inducing oxidative stress^32^, which may be crucial for its pathogenesis. Moreover, based on the fact that liver is a central site of metabolism and is associated with certain cellular factors leading to virus clearance, it is an important organ to explore the host mechanisms that are involved in disease pathophysiology. Therefore, we investigated if the liver would be a potential site for MAYV replication in BALB/c mice model and if its multiplication in this organ may have culminated in unbalanced redox homeostasis and tissue damage, improving the virus pathogenesis.

Here we revealed that the disease caused by MAYV in 3–4-week-old BALB/c mice was self-resolving with high viremia at 1 and 3 d.p.i and detection of neutralizing antibodies in post-infection late time (7 and 10 d.p.i). Infected animals had a lower percentage of weight gain on Days 1 and 3 p.i. and weight recovery was observed from 7 d.p.i. The observed 100% survival rate in infected animals could be explained by the age of mice at the time of infection, when the innate immune system is already well established, contributing to mice overcome lethality.

Weise et al.^44^ inoculated adult CD1 (28-day-old) to develop a live-attenuated MAYV vaccine candidate as a potential virulence model. Mice were infected subcutaneously with 10^5^ p.f.u. of wtMAYV and all mice survived the infection. They observed that wtMAYV-infected mice lost weight initially and recovered it in later times p.i.; however, when adult A129 mice disabled in type 1 interferon receptors were infected on the left footpad with 10^4^ p.f.u. of wtMAYV, the animals presented significant weight loss, a peak viremia titre at Day 2 p.i., and all animals died by Day 5.

In our study, infectious viral particles were recovered in the liver of infected animals on Days 1, 3 and 7 p.i., reinforcing that the liver is a site for MAYV replication. The increase in serum ALT/AST levels in infected animals at all time-points confirmed changes in their liver function. Furthermore, the histological examination of liver tissues of MAYV-infected mice revealed significant increase in the inflammatory infiltrate on Days 1, 3 and 7 p.i. Consistent with our study, Dupuis-Maguiragaet al.^45^ demonstrated a mouse model of CHIKV dissemination, in which the blood would carry the virus to the target organs such as liver, muscle and joints. In these tissues, the infection was associated with infiltration of inflammatory cells mainly during viral replication, leading to pathological events associated with tissue infection. Another study reported the presence of necrotic foci with infiltration of polymorphs and lymphocytes, proliferation of Kupffer cells and presence of apoptotic cells in the liver of new-born Swiss albino mice infected with CHIKV^23^.

Furthermore, we investigated whether MAYV infection in liver of BALB/c mice could alter the redox homeostasis. Therefore, we analysed malondialdehyde (MDA) and carbonyl protein as indicators of oxidative damage in lipids and proteins, respectively. MDA is an aldehyde generated by the free radicals action on fatty acids of the cell membranes^46^, and carbonyl protein is a product from oxidation or carbonylation of proteins, which may cause change of protein function^47^. A significant increase was observed in both biomarkers at all time-points, even after viral clearance from the blood and liver.

Moreover, we also evaluated whether MPO activity was altered in the liver by viral infection as an indirect index of neutrophil activation. When neutrophils are activated, the release of MPO occurs in the phagosome and extracellular compartment. Concomitantly, the enzyme NADPH oxidase acts as a source of hydrogen peroxide radicals (H_2_O_2_), which in turn functions as a cofactor in the generation of oxidants by MPO. These oxidants generated play a pivotal role in microbial death and viral inactivation^48^; however, the excessive generation of these reactive species has been associated with tissue damage due to the accumulation of peroxidase-mediated oxidative damages. These factors lead to the progression of various diseases and particularly pathologies related to inflammatory damage^49^. In our study, an increase in the MPO activity was observed in the liver of MAYV-infected mice at all times post-infection.

The glutathione is the principal non-protein thiol which has been shown by its antioxidant role, maintaining a reductive intra-cellular environment^50,51^. Thus, we measured the hepatic content of T-GSH as well as the reduced and oxidized forms. At early times p.i., T-GSH and GSH contents decreased in the MAYV-infected groups comparing with control groups (Table 1), while GSSG levels increased. Also, we observed GSH/GSSG ratio reduction, which reiterates an evidence of oxidative stress, since it indicates more H_2_O_2_ being produced and more GSH being oxidized in GSSG in order to detoxify ROS.

Collectively, with the increase of stress biomarkers, the MPO activity enhanced, whereas GSH/GSSG ratio observed in our study decreased, confirming here the hepatic oxidative stress induced by MAYV infection. Using the same markers of oxidative stress (MDA, carbonyl protein and GSG/GSSG ratio), we have been confirmed oxidative stress caused by MAYV infection *in vitro*^32^. Similarly, in neuroblastoma SH-SY5Y cells, CHIKV infection causes oxidative stress exclusively by the increase in MDA levels and decrease in the GSH intra-cellular level^52^.

Moreover, since antioxidant system is implicated with oxidative stress^53^, we evaluated SOD and CAT activities as antioxidant enzymatic components. SOD are metalloenzymes that protect the targets of the superoxide anion (O_2_^•−^) attack, converting O_2_^•−^ to H_2_O_2_ as the first line of the enzyme defence system^54^, while CAT is expressed in all major organs of the body, specifically in the liver, kidneys, and erythrocytes and converts H_2_O_2_ to water and oxygen^55^. In general, the activity of these enzymes in the liver of the infected mice decreased at 1, 3 and 7 d.p.i. Nevertheless, at 10 d.p.i. both enzymes were increased. *In vitro*, MAYV infection increased SOD activity at 6, 15 and 24 hours p.i., as well as increased CAT activity at 15 hours p.i. Although increased activity of SOD and CAT has been reported in *in vitro* MAYV infection, here we observe a decrease in the activity of these enzymes soon after the infection. Various studies have shown different types of changes in the SOD and CAT enzyme levels after viral infections^28^. An explanation for these discrepancies could be because oxidative stress can activate by several multistep mechanism, for example the Nrf2 transcription factor, which controls the antioxidant system involved in the redox metabolism^24^. Thus, in redox biology, cell, tissue and temporal variations may be crucial for the physiological functions of antioxidant enzymes during MAYV infection.

Commonly, viruses affect the cellular redox causing homeostasis imbalance^28^; however, the effect of infection on the antioxidant defences depends on the virus, cell type and time of infection. Dhanwani et al.^56^ reported CAT activity decrease in liver of new-born mice infected with CHIKV at 10 d.p.i., leading to liver injury and apoptosis, which evidenced the stress response in the CHIKV pathogenesis. Furthermore, DENV 2-infected mice revealed MDA and GSSG/GSH ratio increase and CAT/SOD activity decrease in serum and liver^57^, indicating the exogenous GSH as a promising therapeutic agent on the oxidative damage prevention.

Regarding viruses that cause known hepatic pathology, it has already been demonstrated that Hepatitis B virus infection causes a reduction in the cytoplasmic SOD1 and increased levels of MDA^58^. Moreover, on Hepatitis C virus infection, have been reported the antioxidant defence mechanisms modified by the virus, for example, changes on gene expression of SOD and CAT, yielding a cellular oxidative imbalance^59^. In Respiratory syncytial virus (RSV) infection, was demonstrated a decreased in SOD, CAT and glutathione peroxidase (GPx) activities, in murine lungs at 1, 3, 5 d.p.i., in which SOD and GPx levels returned to normal at Day 9^35^. Besides, another study also demonstrated changes in the antioxidant defence on Rift Valley Fever virus infection^60^. They observed that infected cells had an early decrease in SOD1 expression with evident oxidative stress.

Thus, a large amount of evidence indicates that oxidative stress is crucial in viral diseases, contributing to viral pathogenesis. This is the first study to report a model in the development of oxidative stress and liver damage at different times after infection by MAYV in which the infection is able to induce high levels of oxidative stress biomarkers and to modulate the antioxidant system. Although conclusions about specific oxidative pathways that contribute to MAYV pathogenesis remain uncertain, this study makes feasible an effective approach to analyze the hepatic disease of MAYV infection and may clarify some early mechanisms that are operating in the host following exposure, besides contributing to future therapeutic approaches in MAYV infection, specifically those based on antioxidant compounds.

## Methods

### Cell culture and virus

Vero (African green monkey kidney cell line) were maintained in Dulbecco’s Modified Eagle’s Medium—DMEM (Cultilab, Brazil), supplemented with 5% foetal bovine serum (FBS; Cultilab, Brazil) in a humidified incubator at 37 °C with 5% CO_2_. The MAYV strain (AC) was originally isolated from a human with Mayaro fever in Acre (Pará, Brazil) and was kindly provided by Professor Maurício Lacerda Nogueira (Faculty of Medicine of São José do Rio Preto/FAMERP/SP). Seed stocks of virus were amplified 2–3 times in Vero cells, and virus pools were aliquoted and stored at −80 °C. Briefly, cells in the 6-well plates were infected with 10-fold virus dilutions with DMEM containing 1% FBS/0.8% carboxymethylcellulose and incubated for 2 days at 37 °C. Cells were fixed with 4% formaldehyde and the plaques were revealed. The virus titre was 10^7^ p.f.u./ml.

### Mouse infection

BALB/c mice were bred and maintained at the Federal University of Ouro Preto (UFOP- Minas Gerais, Brazil). Animal experimentation was approved by the regulations of Animal Ethics Committee (CEUA) of UFOP according to the institutional guidelines, approval number 2016/50. About 36 mice 3–4-week old were inoculated by sub-cutaneous injection with 10^5^ p.f.u. of MAYV and 36 animals were sham inoculated using the same volume of control medium. About four groups containing 18 animals each (9 infected animals and 9 uninfected controls) were daily weighed and monitored during the course of experiment. After 1, 3, 7 and 10 days post-infection (d.p.i.), animals were anesthetised with ketamine and xylazine, and euthanised by exsanguination. Blood samples were collected and centrifuged to obtain serum and to determine viremia, neutralizing antibodies and AST/ALT biomarkers. The livers were removed, and a fragment was fixed in 10% neutral-buffered formalin for histological analysis and the remainder was immediately stored at −80 °C for subsequent analysis.

### Plaque assays for viremia and liver viral load

Plaque assays were performed on Vero cells in six well plates as afore mentioned in the serum and liver samples from infected animals at all tested time points. The livers were macerated in DMEM 0% FBS (Cultilab, Brazil) and centrifuged at 2,000 *g* for 3 min at 4 °C. The supernatant was collected and the viral titre was determined. Titres were expressed as p.f.u. per milliliter of serum or gram of liver. Plaque assays were performed in triplicate for each animal.

### Virus neutralization

Mouse antibody response was tested in the serum of infected mice by Virus Neutralization Test (VNT) using Vero cells and was performed in duplicate. Serum of infected mice (14 µL) was inactivated at 56 °C for 3 min. Subsequently, the serum was serially diluted in base 2 (1:80 to 1:10,240) and was incubated with 100 p.f.u. of MAYV for 1 h at room temperature and was then used to infect Vero cells in 96-well plates. The plates were incubated at 37 °C and examined microscopically. After 72 h, wells were fixed in 10% formaldehyde and stained. The neutralizing antibody titre was calculated and expressed as the reciprocal serum dilution reducing 50% of the cytopathic effect as compared with the virus control. The titre was expressed in neutralizing units (NU) per milliliter of the serum.

### Study of hepatic function markers

Serum levels of AST and ALT were measured to determine the hepatic function using Labtest kits # 52 and 53 (Minas Gerais, Brazil) according to the manufacturer’s instructions.

### Histology

During necropsy, fragments of the liver samples were immediately fixed in 10% neutral-buffered formalin, dehydrated by immersion in a series of ethanol dilutions in water (80%, 90% and 95%) and then in absolute ethanol. Eventually, the tissues were paraffin embedded, sectioned, and stained with hematoxylin and eosin (H&E) at the Laboratory of Immunopathology (UFOP, Brazil). Morphometric measurements of the inflammatory cells in liver sections (25 sections/animal) were performed using a light microscope (Leica DM5000B) and were analysed using Leica QwinV3 Image Processing and Analysis Software (Germany).

### Measurement of lipid peroxidation product

The level of thiobarbituric acid reactive substances (TBARS) was estimated in 20 mg of liver samples using QuantiChromTM TBARS Assay Kit (DTAB-100, BioAssay Systems, USA). At 1, 3, 7 and 10 d.p.i., in liver of the control and infected mice, supernatants were used, and the assay was performed following the manufacturer’s recommendations. The TBARS level was calculated as the concentration of MDA equivalent participating in the reaction in μM.

### Measurement of the protein carbonyl content

The protein carbonyl levels were determined according to the method described^61^. About 60 mg of liver was homogenized in 600 μl of 20 mM phosphate buffer (pH 6.7) and the homogenate was centrifuged at 10,000 *g* for 15 min at 4 °C. At 1, 3, 7 and 10 d.p.i., in the livers of control and infected mice, the protein carbonyl content was measured derivatising the protein carbonyl with 2,4-dinitrophenylhydrazine (DNPH), which resulted in the generation of the dinitrophenyl (DNP) hydrazone product. The absorbance of the samples was observed at 370 nm. The concentration of the DNPH-derivatised proteins was calculated using a molar absorption coefficient of 22,000 M^−1^cm^−1^. The results were expressed in nmol of DNPH incorporated/mg of protein. The total protein content was determined according to the method described by Bradford using bovine serum albumin (BSA) as the standard.

### Myeloperoxidase (MPO) activity

The MPO activity was measured using 3,3′,5,5′-tetramethylbenzidine (TMB), hexadecyltrimethylammonium bromide (HTAB), hydrogen peroxide (H_2_O_2_) and sodium acetate buffer (NaOAc). Initially, 100 mg of the liver tissue was centrifuged with 1 ml of HTAB. Thus, 75 μl of the supernatant was incubated with 5 μl of TMB for 5 min at 37 °C. The mixture was incubated with 50 μl H_2_O_2_ for 10 min at 37 °C. Sequentially, 125 μl of sodium acetate buffer was added. The absorbance was read at 630 nm. The enzymatic activity was expressed as U/mg of total protein.

### Determination of the total, reduced and oxidized glutathione content and GSH/GSSG ratio

Total glutathione content of samples was measured by the glutathione reductase-DTNB (5,5′-dithiobis-(2-nitrobenzoic acid) recycling assay as proposed by Griffith^62^. In this assay, 100 mg of liver homogenate supernatant was used (nine control samples and nine infected samples at each time analysed). The dosage and absorbance of the samples were read in an ELISA plate reader at 412 nm. The glutathione content was expressed as µmol/ml. For oxidized glutathione (GSSG) measurement, 2-vinylpyridine was added to the sample with TEA reagent, which was incubated at room temperature for 1 h and assayed for GSSH concentration. The concentration of reduced glutathione (GSH) was obtained by subtracting the total concentration of the oxidized glutathione. The GSH/GSSG ratio was calculated.

### Biochemical assay for the SOD and CAT activities

To determine the total SOD activity, a biochemical assay was used based on the spectrophotometric method proposed by Marklund and Marklund^63^, which uses inhibition of auto-oxidation of Pyrogallol, whose colour intensity can be determined at 570 nm. Fragments of 20 mg of liver from control and infected animals were homogenized in phosphate buffer (50 mM, pH 7.0) and the supernatant was used. One unit of the enzyme SOD (U SOD) was defined as the amount of enzyme that reduces the auto-oxidation rate of pyrogallol by 50%. The results were expressed in U SOD/mg of protein. To measure the CAT activity, we used the ECAT-100 kit (BioAssay Systems, USA), which directly measures the breakdown of H_2_O_2_ using a redox dye. Fragments of 10 mg of liver from the control and infected animals were homogenized and centrifuged at 12,000 *g* at 4 °C and the supernatant was used for the assay. The absorbance was observed at 570 nm wavelength.

### Statistical analysis

The data were analysed using the GraphPad Prism 6.0 software and expressed as the mean ± standard error (SEM) of nine samples per group. For the parametric data, Student’s t-test at 95% confidence was used to determine the difference level between the MAYV-infected and the uninfected mice, where *p < 0.05, **p < 0.01 and ***p < 0.001. One-way ANOVA was used to compare the virus titres in serum and liver between different experimental groups and two-way ANOVA was used to compare the inflammatory cells in the liver among the different experimental groups. When the changes were significant, the Bonferroni post-test was performed.

## Data Availability

The experimental data used to support the findings of this study are included in the article and the readers can have access to it through the article content. Raw data regarding the findings and any other information can be requested by the reader to the correspondent author of the paper via e-mail.
